# Risk factors and predictive model for nosocomial infections by extensively drug-resistant *Acinetobacter baumannii*

**DOI:** 10.3389/fcimb.2024.1475428

**Published:** 2024-09-30

**Authors:** Jingchao Shi, Xiaoting Mao, Jianghao Cheng, Lijia Shao, Xiaoyun Shan, Yijun Zhu

**Affiliations:** ^1^ Department of Clinical Laboratory, Affiliated Jinhua Hospital, Zhejiang University School of Medicine (Jinhua Municipal Central Hospital), Jinhua, Zhejiang, China; ^2^ Department of Clinical Laboratory, Jinhua Maternal and Child Health Care Hospital, Jinhua, Zhejiang, China; ^3^ Department of Clinical Laboratory, Zhejiang Cancer Hospital, Hangzhou, Zhejiang, China

**Keywords:** extensively drug-resistant *Acinetobacter baumannii*, risk factors, predictive model, LASSO regression, nomogram

## Abstract

**Background:**

Extensively drug-resistant Acinetobacter baumannii (XDRAB) has become a significant pathogen in hospital environments, particularly in intensive care units (ICUs). XDRAB’s resistance to conventional antimicrobial treatments and ability to survive on various surfaces pose a substantial threat to patient health, often resulting in severe infections such as ventilator-associated pneumonia (VAP) and bloodstream infections (BSI).

**Methods:**

We retrospectively analyzed clinical data from 559 patients with XDRAB infections admitted to Jinhua Central Hospital between January 2021 and December 2023. Patients were randomly divided into a training set (391 cases) and a testing set (168 cases). Variables were selected using Lasso regression and logistic regression analysis, and a predictive model was constructed and validated internally and externally. Model performance and clinical utility were evaluated using the Hosmer-Lemeshow test, C-index, ROC curve, decision curve analysis (DCA), and clinical impact curve (CIC).

**Results:**

Lasso regression analysis was used to screen 35 variables, selecting features through 10-fold cross-validation. We chose lambda.1se=0.03450 (log(lambda.1se)=-3.367), including 10 non-zero coefficient features. These features were then included in a multivariate logistic regression analysis, identifying 8 independent risk factors for XDRAB infection: ICU stay of 1-7 days (OR=3.970, 95%CI=1.586-9.937), ICU stay >7 days (OR=12.316, 95%CI=5.661-26.793), hypoproteinemia (OR=3.249, 95%CI=1.679-6.291), glucocorticoid use (OR=2.371, 95%CI=1.231-4.564), urinary catheterization (OR=2.148, 95%CI=1.120-4.120), mechanical ventilation (OR=2.737, 95%CI=1.367-5.482), diabetes mellitus (OR=2.435, 95%CI=1.050-5.646), carbapenem use (OR=6.649, 95%CI=2.321-19.048), and β-lactamase inhibitor use (OR=4.146, 95%CI=2.145-8.014). These 8 factors were used to construct a predictive model visualized through a nomogram. The model validation showed a C-index of 0.932 for the training set and 0.929 for the testing set, with a Hosmer-Lemeshow test p-value of 0.47, indicating good calibration. Furthermore, the DCA curve demonstrated good clinical decision-making performance, and the CIC curve confirmed the model’s reliable clinical impact.

**Conclusion:**

Regression analysis identified ICU stay duration, hypoproteinemia, glucocorticoid use, urinary catheterization, mechanical ventilation, diabetes mellitus, carbapenem use, and β-lactamase inhibitor use as independent risk factors for XDRAB infection. The corresponding predictive model demonstrated high accuracy and stability.

## 1 Introduction

*Acinetobacter baumannii* is a Gram-negative, non-fermentative bacterium that has emerged as a significant pathogen in hospital environments due to its remarkable ability to survive on various surfaces and its intrinsic resistance to many antibiotics (Shi et al., 2024). This bacterium is one of the most problematic organisms in healthcare-associated infections (HAIs), responsible for severe infections such as ventilator-associated pneumonia (VAP), bloodstream infections (BSI), skin and soft-tissue infections, and bacteremia. These infections often result in ineffective early-stage treatment and increased mortality rates (Fu et al., 2015). A survey of nosocomial infections in tertiary general hospitals in China identified *A. baumannii* as the most common pathogen in HAIs (Jian et al., 2023). Reports of extensively drug-resistant *A. baumannii* (XDRAB) are increasing, with ventilator-associated pneumonia (VAP) and bloodstream infections (BSI) being the most common and having high mortality rates. In recent years, the probability of detecting XDRAB in ICU patients has been increasing (Fang et al., 2023; Fitzpatrick et al., 2022). This pathogen has multiple resistance mechanisms, rendering conventional antimicrobial drugs ineffective. ICU patients often have severe and complex conditions, poor baseline health, and are frequently co-infected with VAP and BSI, leading to a bloodstream infection mortality rate of 40% (Garnacho-Montero et al., 2015). Although studies on XDRAB infection exist, regional variations necessitate early detection of risk factors and effective interventions to reduce mortality, minimize complications, and improve patient prognosis. This article aims to retrospectively analyze clinical data from XDRAB-infected patients to establish a practical infection prediction model using various statistical analyses. Line graphs will be employed to convert complex regression equations into easy-to-read visuals, facilitating the assessment of XDRAB infection risk.

## 2 Meterial and methods

### 2.1 Patient selection

In this study, we retrospectively analyzed the clinical data of patients infected with extensively drug-resistant *Acinetobacter baumannii* (XDRAB) who were hospitalized at Jinhua Central Hospital between January 2021 and December 2023. Based on the parameters to be estimated in this study, we referred to relevant literature to assess the required sample size (Riley et al., 2020). A total of 559 subjects were included, comprising 212 patients in the XDRAB-infected group and 347 patients in the non-XDRAB-infected *Acinetobacter baumannii* (AB)-infected group. All patients were randomly assigned to either the training set (391 cases) or the test set (168 cases) using a 7:3 randomization model by R software. The training set was used to construct the predictive model, while the test set was used for model validation. This retrospective study was conducted in accordance with the Transparent Reporting of a Multivariable Prediction Model for Individual Prognosis or Diagnosis (TRIPOD) guidelines (Collins et al., 2015). The study was approved by the Ethics Committee of Jinhua Central Hospital (Ethical Number: (2024) Lun Audit No. (1)), and all procedures followed ethical guidelines and regulations.

### 2.2 Screening criteria

#### 2.2.1 Inclusion criteria

1. Patients hospitalized at Jinhua Central Hospital from January 2021 to December 2023.
2. Patients meeting the Centers for Disease Control and Prevention (CDC) criteria for *A. baumannii* infection (Horan et al., 2008).
3. Types of clinically infected AB specimens include sputum, bronchoalveolar lavage fluid, urine, pleural fluid, ascites, cerebrospinal fluid, blood, wound secretions, and puncture fluid.
4. Only the first AB infection in each patient was included.
5. XDRAB is defined as a strain of *A. baumannii* susceptible to only one or two drugs with potential antifusobacterial activity (mainly polymyxins and tigecycline).

#### 2.2.2 Exclusion criteria

1. Contaminated samples or duplicate strains from the same patient.
2. Patients who were infected with pathogens other than AB or who were colonized with AB without clinical infection.
3. Patients with AB infection detected within 48 hours of admission, patients transferred out of the hospital within 48 hours of admission, patients who died, or patients who abandoned treatment.
4. Patients with significant missing clinical data.

### 2.3 Data collection

In this study, potential predictive variables were collected and analyzed with reference to relevant literature. These variables included age, sex, smoking history, drinking history, ICU stay, hypoproteinemia, use of glucocorticoids, surgical history, central venous cannulation (CVC), tracheal intubation, mechanical ventilation, urinary catheterization, other invasive procedures, hemodialysis, shock, and various blood parameters (including white blood cell count, neutrophil count, lymphocyte count, neutrophil-to-lymphocyte ratio (NLR), red blood cell count, hemoglobin, platelet count, C-reactive protein, calcitonin, and interleukin-6). Additionally, the history of chronic pneumonia, cerebrovascular disease, cardiovascular disease, diabetes mellitus, hypertension, and antibiotic use were recorded. All potential risk factors were present prior to XDRAB infection.

ICU admission times were divided into three intervals:

1. No ICU admission

2. ICU admission time of 1 to 7 days

3. ICU admission time greater than 7 days

These intervals were calculated from the day of admission to the first AB detection. Hypoproteinemia was defined as serum albumin below 30 g/L. Antibiotic usage was defined by the type of antimicrobial drugs used before contracting AB, including aminoglycosides (e.g., tobramycin, amikacin, gentamicin), carbapenems (e.g., imipenem, meropenem), quinolones (e.g., levofloxacin, ciprofloxacin), beta-lactamase inhibitors (e.g., piperacillin-tazobactam, cefoperazone -sulbactam), and broad-spectrum cephalosporins (e.g., ceftazidime, ceftriaxone, cefepime).

### 2.4 Statistical analysis

In this study, SPSS 22.0 software was used for statistical analysis, and R 4.3.1 software was employed to screen characteristic variables, construct the prediction model, and validate it. As the study is retrospective, some continuous variables had individual missing values. The “VIM” package in R was used to visualize the distribution of missing values. If a potential risk factor had more than 10% missing values, it was excluded. For factors with less than 10% missing values, the “MICE” package in R was used to perform multiple imputation.

For continuous variables, normality was assessed using the Kolmogorov-Smirnov test. Variables conforming to normal distribution were expressed as mean ± standard deviation and analyzed using the independent samples t-test. Non-normally distributed variables were expressed as median (interquartile range) and analyzed using the Mann-Whitney U-test. Categorical data were described using frequencies and percentages and compared using the χ2 test or Fisher’s exact test.

To reduce the dimensionality of the data, address multicollinearity-induced model overfitting, and enhance the model’s generalizability and applicability, we employed the Least Absolute Shrinkage and Selection Operator (Lasso) regression algorithm, which penalizes the coefficients of all variables in the model (Simon and Tibshirani, 2012). We used the “glmnet” package in R to perform Lasso regression for variable selection, utilizing 10-fold cross-validation to determine the appropriate lambda value. The lambda value determines the degree of penalization for the regression coefficients, the larger the lambda, the greater the penalization, forcing non-characteristic variables to be precisely zero. We selected the non-zero variables corresponding to the lambda.1se value in the cross-validation for inclusion in the multivariable logistic regression analysis to identify independent risk factors for XDRAB infection. Based on the regression coefficients, we constructed a visual predictive nomogram using the “rms” package in R. The predictive probability of XDRAB infection was calculated by summing the scores of each indicator in the nomogram.

The model was internally validated using 1000 bootstrap samples from the training set and externally validated using the test set data. The Hosmer-Lemeshow test was employed to assess model calibration. A p-value > 0.05 indicated good model fit, while a p-value < 0.05 indicated poor model fit. The C-index (Harrell et al., 1996) and the Area Under the Curve (AUC) of the Receiver Operating Characteristic (ROC) curve were used to quantify the model’s discriminatory power and prediction accuracy. A C-index of 0.50–0.70 indicated low accuracy, 0.71–0.90 indicated moderate accuracy, and greater than 0.91 indicated high accuracy. An AUC less than 0.5 indicated poor model discrimination, 0.5–0.7 indicated low discrimination, 0.7–0.85 indicated good discrimination, and greater than 0.85 indicated excellent discrimination. To assess the clinical utility of the model, Decision Curve Analysis (DCA) (Vickers and Elkin, 2006) and Clinical Impact Curve (CIC) analyses (Kerr et al., 2016) were performed.

## 3 Results

### 3.1 Analysis of XDRAB and NXDRAB patient data

**Figure 1:**
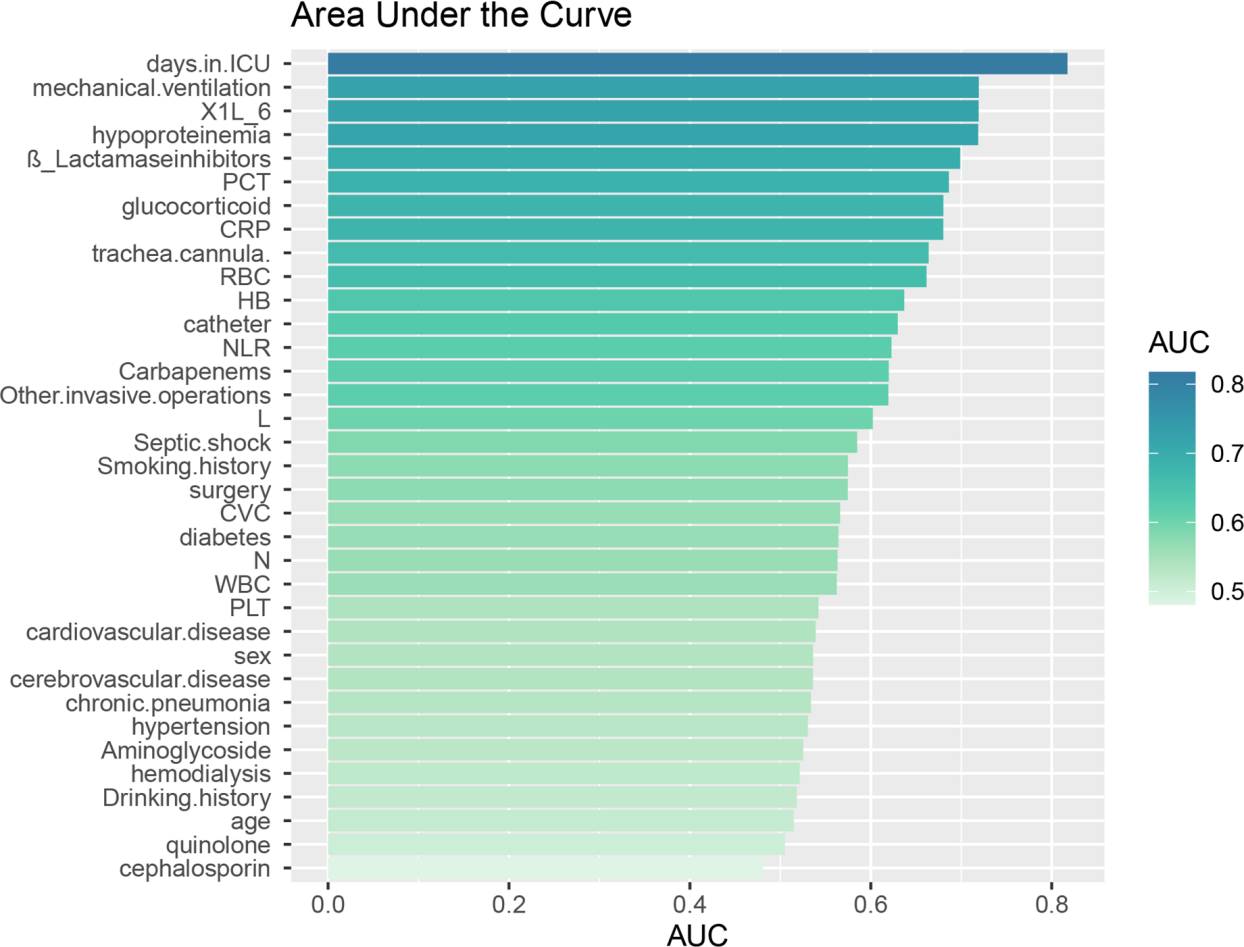
Area under the ROC curve (AUC) for individual data. The Area Under the ROC Curve (AUC) represents the diagnostic accuracy of individual data as evaluated by the model test.

A total of 559 patients were included in this study, with 212 patients in the XDRAB group and 347 patients in the non-XDRAB (NXDRAB) group. Comparison of baseline data showed significant differences between the XDRAB and NXDRAB groups in terms of smoking history, chronic pneumonia history, cardiovascular disease history, diabetes mellitus, duration of ICU stay, hypoproteinemia, glucocorticoid use, surgery, central venous catheterization, endotracheal tube placement, mechanical ventilation, urinary catheterization, other invasive procedures, shock, white blood cell counts (including neutrophils and lymphocytes), erythrocyte counts, hemoglobin, procalcitonin, interleukin-6, CRP levels, and the use of carbapenem and β-lactamase inhibitor medications (*P*<0.05). See **Table 1** and **Figure 1**.

**Table 1:** Clinical characteristics of patients with XDRAB and NXDRAB.

Characteristics	Total patients N=559	NXDRAB N=347	XDRAB N=212	*P*-value
Age, Median (IQR)	69 (58, 77)	69 (58, 77)	70 (57.75, 79)	0.557
Sex, N (%)				0.094
male	373 (67)	222 (64)	151 (71)	
women	186 (33)	125 (36)	61 (29)	
Smoking history, N (%)				< 0.001
there are	109 (19)	48 (14)	61 (29)	
not have	450 (81)	299 (86)	151 (71)	
Drinking history, N (%)				0.277
there are	77 (14)	43 (12)	34 (16)	
not have	482 (86)	304 (88)	178 (84)	
Chronic pneumonia, N (%)				0.038
there are	82 (15)	42 (12)	40 (19)	
not have	477 (85)	305 (88)	172 (81)	
Cerebrovascular disease, N (%)				0.056
there are	120 (21)	65 (19)	55 (26)	
not have	439 (79)	282 (81)	157 (74)	
Cardiovascular disease, N (%)				0.015
there are	81 (14)	40 (12)	41 (19)	
not have	478 (86)	307 (88)	171 (81)	
Diabetes, N (%)				< 0.001
there are	111 (20)	52 (15)	59 (28)	
not have	448 (80)	295 (85)	153 (72)	
Hypertension, N (%)				0.19
there are	290 (52)	172 (50)	118 (56)	
not have	269 (48)	175 (50)	94 (44)	
Length of ICU stay,N (%)				< 0.001
Not admitted to ICU	318 (57)	273 (79)	45 (21)	
1~7 days	80 (14)	43 (12)	37 (17)	
Greater than 7 days	161 (29)	31 (9)	130 (61)	
Hypoproteinemia, N (%)				< 0.001
there are	249 (45)	97 (28)	152 (72)	
not have	310 (55)	250 (72)	60 (28)	
Glucocorticoids, N (%)				< 0.001
there are	252 (45)	109 (31)	143 (67)	
not have	307 (55)	238 (69)	69 (33)	
Surgery, N (%)				< 0.001
there are	241 (43)	130 (37)	111 (52)	
not have	318 (57)	217 (63)	101 (48)	
Central venous catheterization, N (%)				< 0.001
there are	86 (15)	36 (10)	50 (24)	
not have	473 (85)	311 (90)	162 (76)	
Tracheal intubation, N (%)				< 0.001
there are	229 (41)	99 (29)	130 (61)	
not have	330 (59)	248 (71)	82 (39)	
Mechanical ventilation, N (%)				< 0.001
there are	267 (48)	108 (31)	159 (75)	
not have	292 (52)	239 (69)	53 (25)	
Catheter, N (%)				< 0.001
there are	229 (41)	108 (31)	121 (57)	
not have	330 (59)	239 (69)	91 (43)	
Other intrusive operations, N (%)				< 0.001
there are	194 (35)	89 (26)	105 (50)	
not have	365 (65)	258 (74)	107 (50)	
Hemodialysis, N (%)				0.048
there are	30 (5)	13 (4)	17 (8)	
not have	529 (95)	334 (96)	195 (92)	
Shock, N (%)				< 0.001
there are	65 (12)	18 (5)	47 (22)	
not have	494 (88)	329 (95)	165 (78)	
Leukocytes (×10^9^ /L),Median (IQR)	7.71 (5.3, 11.14)	7.11 (4.89,11.46)	8.09 (5.95,10.95)	0.013
Neutrophils (×10^9^ /L),Median (IQR)	5.87 (3.96,9.44)	5.6 (3.55,9.54)	6.46 (4.59, 9.15)	0.012
Lymphocytes (×10^9^ /L),Median (IQR)	0.9 (0.57,1.31)	0.96 (0.66,1.39)	0.76 (0.48,1.19)	< 0.001
NLR, Median (IQR)	7.66 (4.45, 11.5)	6.82 (4.18,10.54)	8.92 (5.48,14.33)	< 0.001
Erythrocytes (×10^12^ /L), Median (IQR)	3.45 (3.02, 3.95)	3.65 (3.16,4.07)	3.26 (2.79, 3.6)	< 0.001
Hemoglobin (g/L), Median (IQR)	104 (91,118)	107 (95,120)	98 (85, 110.25)	< 0.001
Platelets (×10^9^ /L),Median (IQR)	193 (124.5, 273.5)	194 (141, 266)	190 (96.5, 299)	0.094
Calcitoninogen, Median (IQR)	0.45 (0.09,1.17)	0.25(0.07,0.62)	0.87 (0.21,2.25)	< 0.001
Interleukin-6, Median (IQR)	27.36 (12.3, 77.4)	20.1 (10.25, 43.6)	58.27 (22.7, 171.48)	< 0.001
CRP, Median (IQR)	20.9 (11.3, 29.86)	18.11 (9.8, 25.17)	29.86 (12.83, 73.16)	< 0.001
Carbapenems, N (%)				< 0.001
there are	75 (13)	15 (4)	60 (28)	
not have	484 (87)	332 (96)	152 (72)	
Cephalosporins, N (%)				0.28
there are	87 (16)	59 (17)	28 (13)	
not have	472 (84)	288 (83)	184 (87)	
Aminoglycosides, N (%)				0.062
there are	51 (9)	25 (7)	26 (12)	
not have	508 (91)	322 (93)	186 (88)	
Quinolones, N (%)				0.792
there are	44 (8)	26 (7)	18 (8)	
not have	515 (92)	321 (93)	194 (92)	
Beta-lactamase inhibitors, N (%)				< 0.001
	247 (44)	101 (29)	146 (69)	
	312 (56)	246 (71)	66 (31)	

XDRAB, Extensively Drug Resistant Acinetobacter baumannii; NXDRAB, Non- Extensively Drug Resistant Acinetobacter baumannii; N, Total number; CRP, C-reactive protein; IQR, interquartile range Beta-lactamase inhibitors: piperacillin/tazobactam + cefoperazone/sulbactam + ticarcillin/clavulanic.

### 3.2 Screening for infection factors

**Figure 2:**
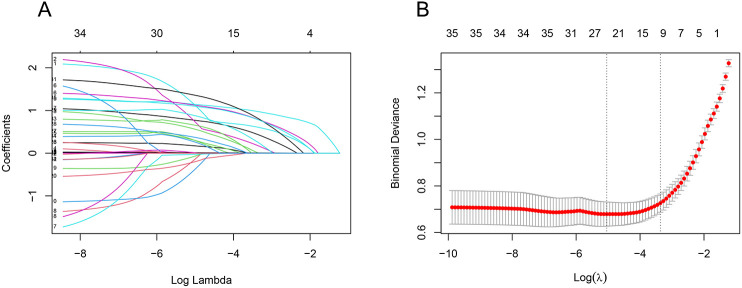
LASSO regression screening feature subset. The Least Absolute Shrinkage and Selection Operator (LASSO) regression was used to screen the feature subset. **(A)** LASSO coefficient curves for 35 variables, showing the coefficient distributions generated for the log(λ) series. **(B)** The suitable parameter (λ) was selected by 10-fold cross-validation, with dashed lines indicating lambda.min and lambda.1se. The left dashed line represents lambda.min, and the right dashed line represents lambda.1se. (lambda.min denotes the value of λ when the model error is smallest, and lambda.1se denotes the value of λ when the model error is within one standard error.) The goal is to include the least number of variables while ensuring a good fit to create the most streamlined prediction model. In this study, lambda.1se was selected as the optimal λ value.

We used LASSO regression to screen 35 variables, performing feature selection through 10-fold cross-validation. As the value of lambda changes in the LASSO algorithm, we tracked how the coefficients (which represent the importance of each predictor) behaved (**Figure 2**). At the point where lambda was at its minimum (lambda.min = 0.0065 [log(lambda.min) = -0.5041]), we identified 24 variables with non-zero coefficients. However, including too many variables can make the model more complex and less generalizable. Therefore, we chose a slightly larger lambda value (lambda.1se = 0.03450 [log(lambda.1se) = -3.367]) that resulted in a more streamlined model with only 10 variables. These 10 key variables included smoking history, length of ICU stay, low protein levels, use of glucocorticoids, urinary catheterization, mechanical ventilation, CRP levels, diabetes, use of carbapenems, and use of β-lactamase inhibitors (**Figure 2**).

### 3.3 Multivariate logistic regression analysis

The outcome variable was XDRAB infection, and the above subset of 10 characteristics were included as independent variables in the multivariate logistic regression analysis. The results showed that an ICU stay of 1 to 7 days (OR = 3.970, 95% CI = 1.586–9.937), ICU stay >7 days (OR = 12.316, 95% CI = 5.661–26.793), hypoproteinemia (OR = 3.261, 95% CI = 1.679–6.291), glucocorticoid use (OR = 2.371, 95% CI = 1.231–4.564), catheterization (OR = 2.148, 95% CI = 1.120–4.120), mechanical ventilation (OR = 2.737, 95% CI = 1.367–5.482), diabetes mellitus (OR = 2.435, 95% CI = 1.050–5.646), carbapenem use (OR = 6.649, 95% CI = 2.321–19.048), and β-lactamase inhibitor use (OR = 4.146, 95% CI = 2.145–8.014) were independent risk factors for the development of XDRAB infection in hospitalized patients. See **Table 2**.

**Table 2:** Multifactor logistic regression analysis.

Characteristics	*β*	S.E	*P*-value	OR	95% CI
smoking history	0.419	0.397	0.291	1.521	0.699~3.311
ICU 1~7 days	1.132	0.422	0.007	3.970	1.586~9.937
ICU > 7 days	2.511	0.397	<0.001	12.316	5.661~26.793
hypoproteinemia	1.179	0.337	<0.001	3.250	1.679 ~6.291
glucocorticosteroid	0.863	0.334	0.010	2.371	1.231 ~4.564
mechanical ventilation	1.007	0.354	0.004	2.737	1.367 ~5.482
urinary catheter	0.764	0.332	0.021	2.148	1.120 ~4.120
CRP	0.009	0.006	0.122	0.992	0.981 ~1.002
diabetes	0.890	0.429	<0.001	2.435	1.050 ~5.646
carbapenems	1.895	0.537	<0.001	6.649	2.321 ~19.048
Beta-lactamase inhibitor drugs	1.422	0.336	<0.001	4.146	2.145 ~8.014

β: regression coefficient; S.E: standard error.

### 3.4 Creation of XDRAB infection prediction nomogram

**Figure 3:**
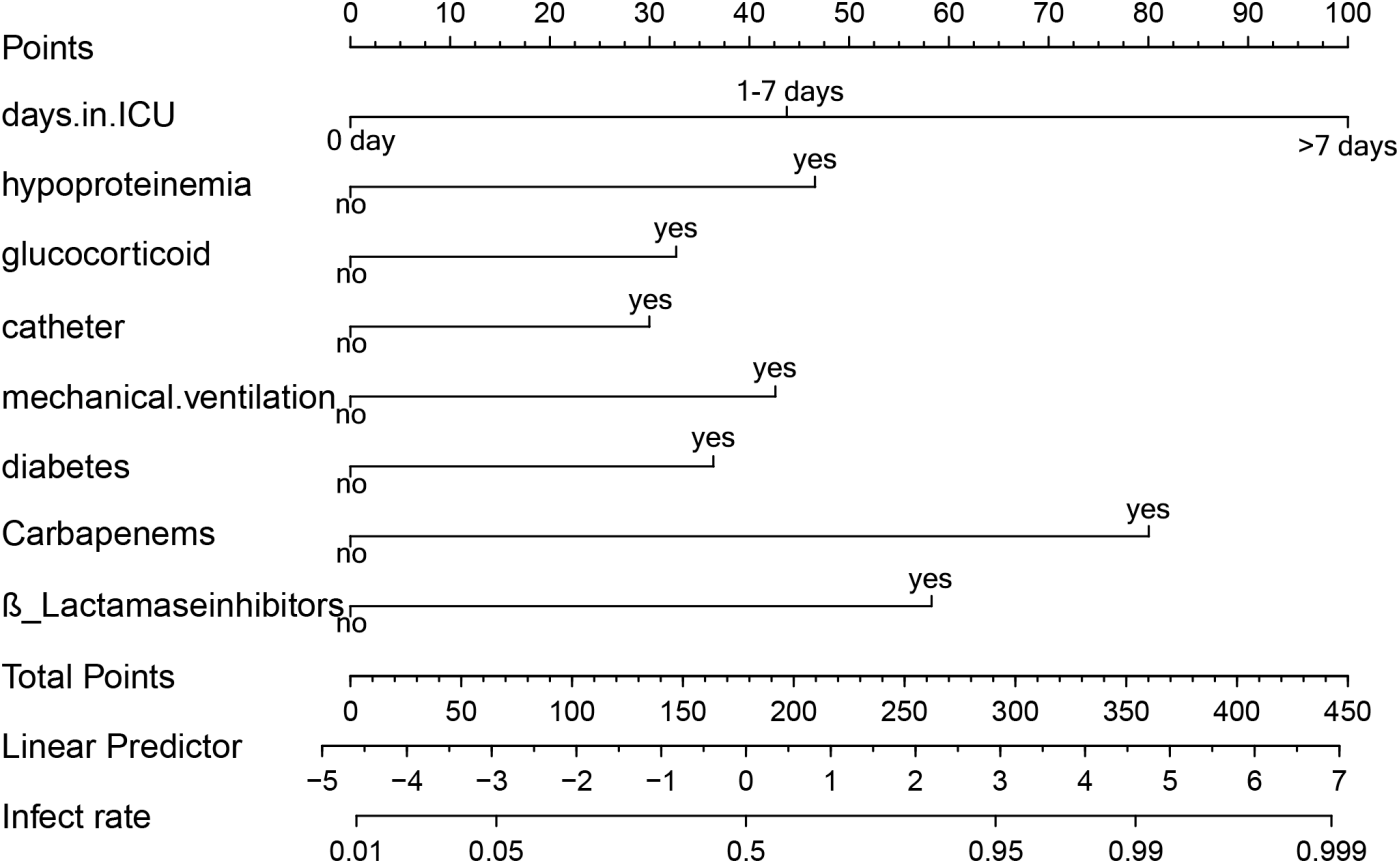
Line graph of predicted risk of XDRAB infection in hospitalized patients. Each item corresponds to an option, which corresponds to the numerical point above. All option values sum up to the final TOTAL POINT, which corresponds to the probability of infection below.

Predictors obtained from the multivariate logistic regression analysis—ICU length of stay, hypoproteinemia, glucocorticoid use, urinary catheterization, mechanical ventilation, diabetes mellitus, carbapenem use, and β-lactamase inhibitor use—were included in the model. Based on the regression coefficients, a visualized prediction nomogram was constructed. The total score was obtained by summing the scores corresponding to each indicator, and the corresponding prediction probability of XDRAB infection was derived (**Figure 3**). For instance, if a patient has been in the ICU for more than 7 days, the corresponding score on the nomogram is 100 points. If the patient has also undergone mechanical ventilation, an additional 42.5 points are added. Further, if the patient has used glucocorticoids and carbapenem antibiotics, these add 32.5 and 80 points respectively. The total score in this case would be 255 points, which correlates with an estimated infection probability of around 82%.

This scoring system allows clinicians to quickly assess the risk level of patients based on their clinical characteristics and make informed decisions about their care.

### 3.5 Validation of predictive models

**Figure 4:**
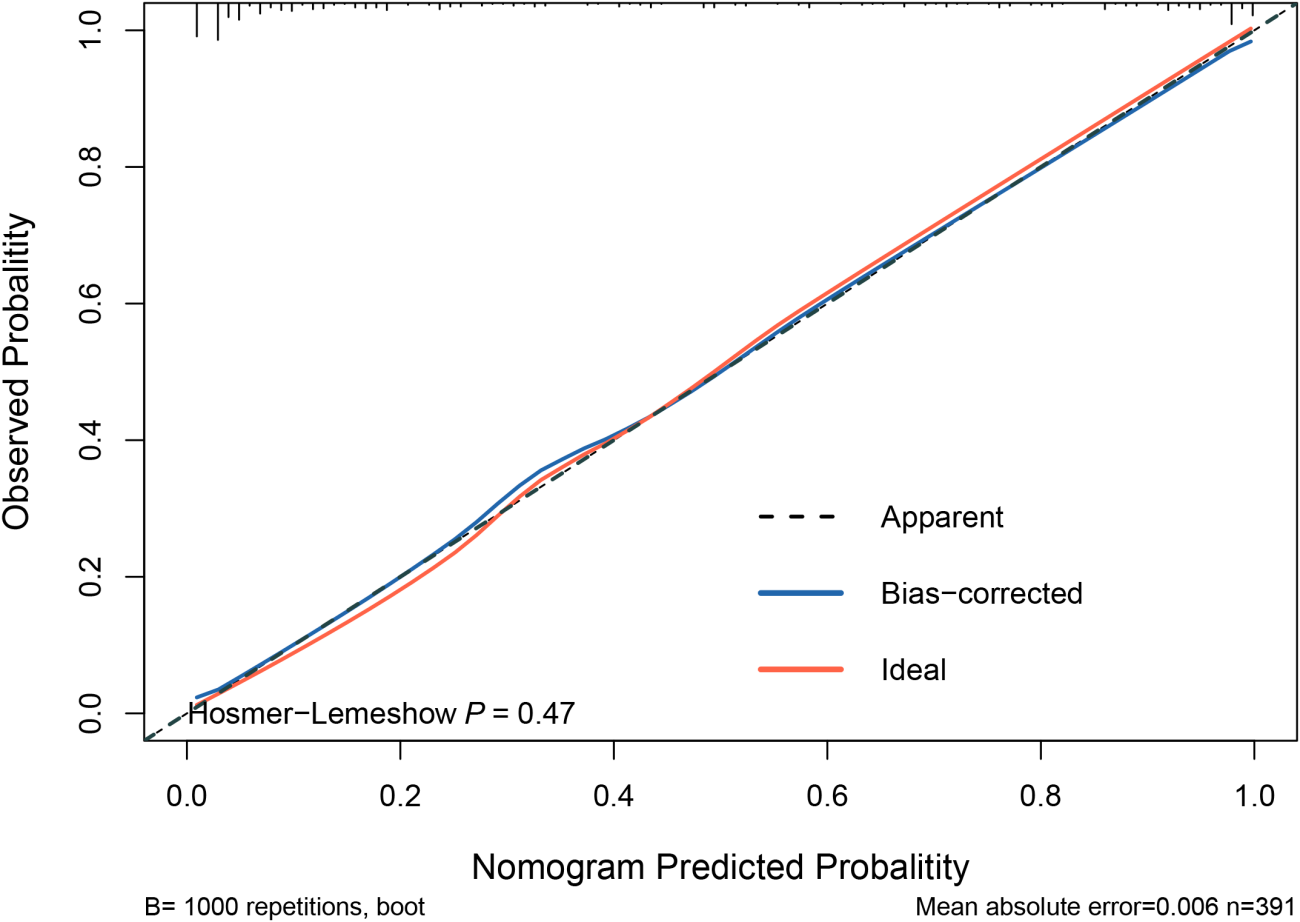
Calibration curves for predictive models. The Hosmer-Lemeshow test yielded a p-value of 0.47, indicating no significant difference between the predicted and actual values of the model. This suggests that the model is well-calibrated. Additionally, the calibration curve closely oscillates around the 45° diagonal line, further demonstrating high calibration accuracy.

**Figure 5:**
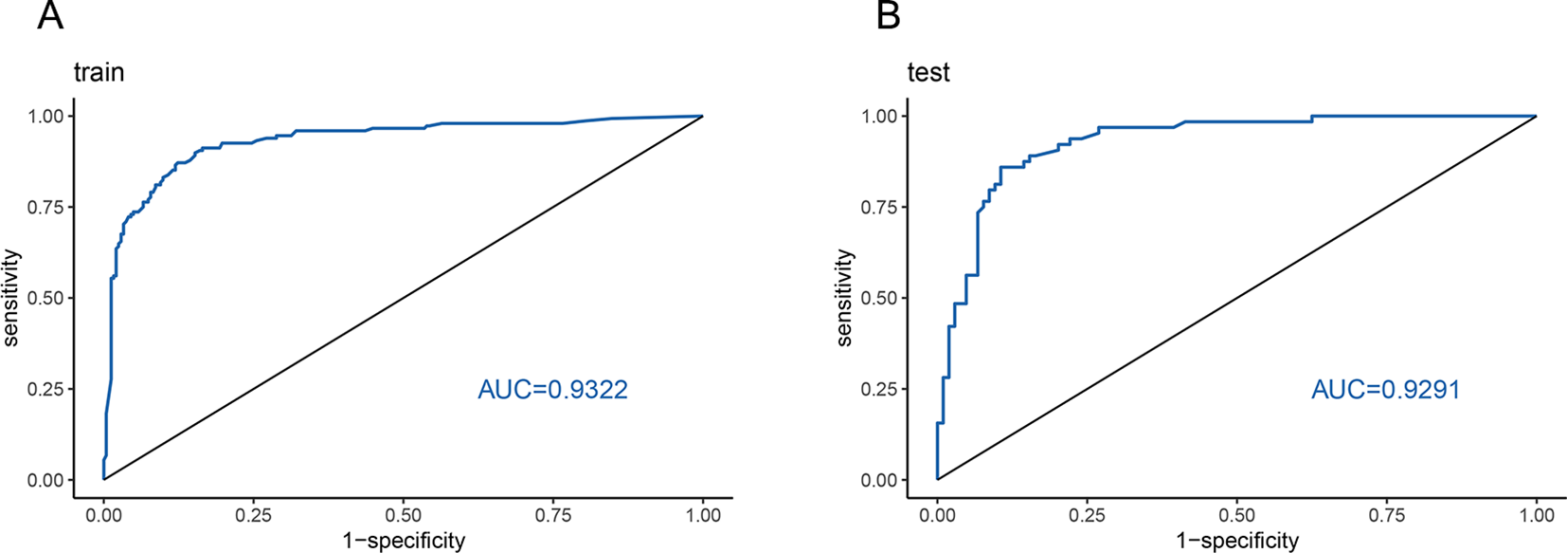
ROC curve for validating the nomogram. **(A)** Training set validation ROC curve with an area under the curve (AUC) of 0.9322. **(B)** Test set validation ROC curve with an AUC of 0.9291.

The model was internally validated using the training set with a C-index of 0.932 and a p-value of 0.47 from the Hosmer-Lemeshow test, indicating no significant difference between the predicted and actual values. The calibration curve oscillated around the 45° diagonal line, showing a high degree of calibration (**Figure 4**). The ROC curves of the training and test sets showed AUCs of 0.932 and 0.929, respectively, indicating good discrimination and consistency (**Figure 5**).

### 3.6 Clinical applications of predictive modeling

**Figure 6:**
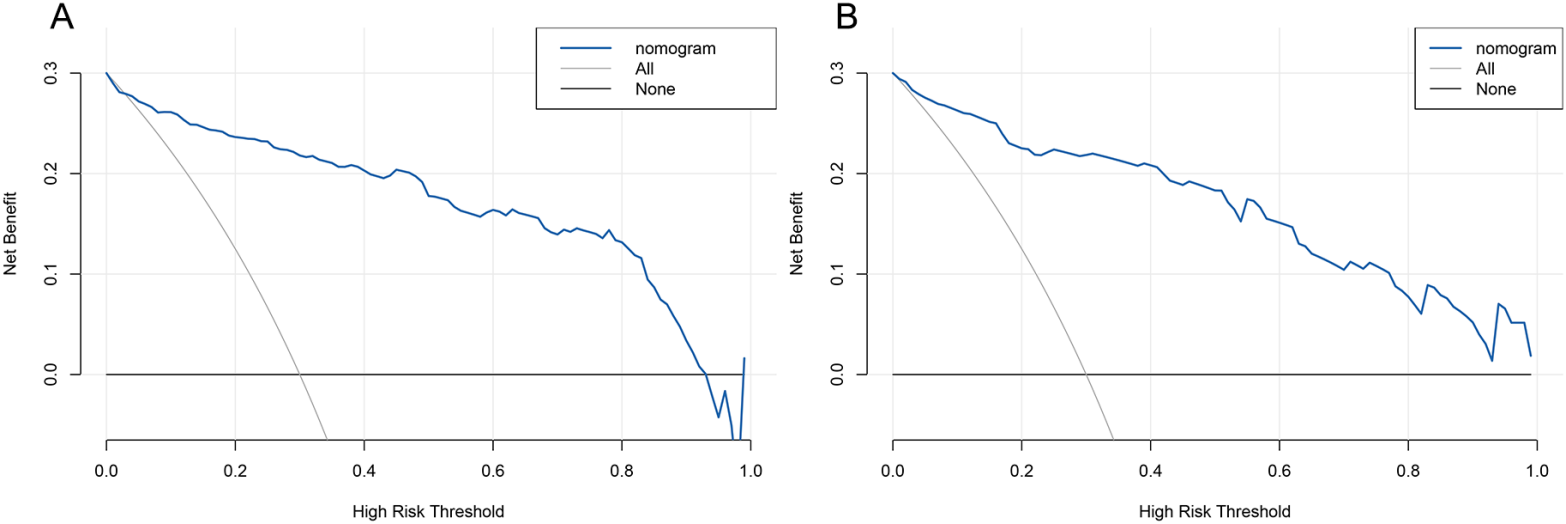
DCA curve for predictive model validation. **(A)** DCA curve for the training set. **(B)** DCA curve for the test set. DCA, Decision Curve Analysis.

**Figure 7:**
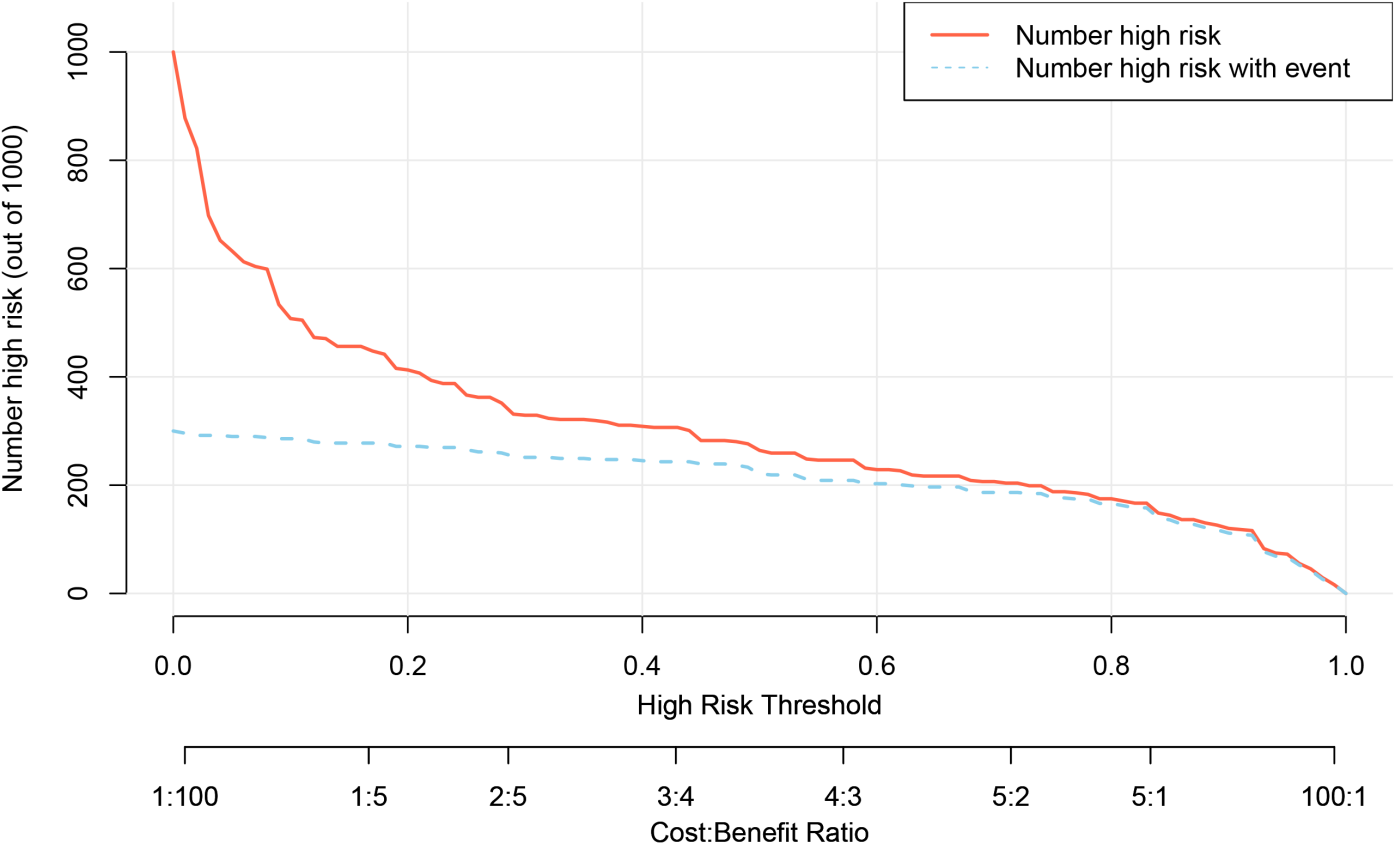
CIC curves for predictive model validation. CIC, Clinical Impact Curve. The horizontal axis (x-axis) represents the threshold probabilities (Pt), which are the decision points where clinicians may consider a patient at high risk for infection. The vertical axis (y-axis) shows the number of patients identified as high risk. The upper curve depicts the total number of patients classified as high risk by the model at each threshold, while the lower curve represents the true positive cases—patients correctly identified as having the infection. The closer the lower curve is to the upper curve, the more accurately the model predicts true positives, minimizing false positives and highlighting its reliability in a clinical setting.

We used Decision Curve Analysis (DCA) and Clinical Impact Curve (CIC) to evaluate the net benefit and clinical reliability of the model. As shown in **Figure 6**, the DCA curves for the validation of the nomogram (A for the training set and B for the test set) are plotted. The horizontal axis represents the threshold probability (Pt), which is the predicted score value at which the probability of a patient contracting XDRAB is Pi (Predicted Probability of Infection). When Pi reaches a threshold Pt, it is classified as positive, and intervention measures are taken. The vertical axis represents the net benefit rate. Both curves (A and B) are far from the extreme sloped lines, indicating better clinical utility. As shown in **Figure 7**, the CIC curves illustrate both the number of individuals the model predicts to be at high risk for XDRAB infection at different probability thresholds and the actual number of true high-risk cases. The close alignment between these curves across various thresholds underscores the model’s strong clinical utility, effectively balancing the identification of true positives with minimizing false positives.

## 4 Discussion

The aim of this study was to investigate potential risk factors for extensively drug-resistant *Acinetobacter baumannii* (XDRAB) infection in hospitalized patients and to develop a prediction model for such infections. XDRAB infections cause prolonged hospitalization, complications, increased patient mortality, and significant financial burden on patients’ families (Zhen et al., 2021). In our study, we used LASSO regression and multivariate logistic regression analyses to identify the most influential factors, thereby reducing confounding bias. The results indicated that ICU length of stay, hypoproteinemia, glucocorticoid use, urinary catheters, mechanical ventilation, diabetes mellitus, carbapenem use, and β-lactamase inhibitor medications were independent risk factors for XDRAB infection in hospitalized patients. Subsequently, we developed a nomogram based on these eight predictors, which has great potential for clinical application in early detection of XDRAB infection.

In our previous survey, the detection rate of XDRAB was highest in ICU wards, accounting for almost 50% of the hospital’s detection rate, making the ICU a “reservoir” of XDRAB. ICU patients often suffer from acute or critical illnesses, many with underlying diseases, rapid progression, and a high risk of disease deterioration. The risk of XDRAB infection increases under high doses of antimicrobials due to low patient mobility, prolonged bed rest, difficulty in cooperating with clinical examinations, and more invasive procedures weakening the body’s protective barriers (Wu et al., 2023). Our study showed that the risk of XDRAB infection increased significantly with ICU stay, particularly for stays longer than seven days. Therefore, early transfer from the ICU to general wards could reduce the risk of XDRAB infection for eligible patients.

We also found that patients with hypoproteinemia were more susceptible to XDRAB. Hypoproteinemia, an indicator of weakness and instability, often leads to lower immunity and poorer physical condition, particularly in ICU patients, increasing the risk of infection (Ushizawa et al., 2016). A study has shown a strong association between hypoproteinemia and death from AB infection (Zhang et al., 2020). The extensive drug resistance of XDRAB, coupled with the low immunity of patients, further elevates the risk of infection.

Prolonged and extensive use of glucocorticoids impairs immune function by reducing the immune response. This suppression of alveolar macrophages leads to increased colonization by respiratory pathogens, toxin accumulation, and a shift in favor of pathogenic bacterial growth (Hogg, 2012). Research has identified steroid use as an independent risk factor for mortality from bacteremia in patients infected with AB (Townsend et al., 2015; Ballouz et al., 2017).

The ICU environment, combined with complex patient conditions, can lead to aerosol contamination from open suctioning operations, increasing the chance of cross-infection. AB’s ability to produce biofilm enhances its colonization, allowing it to persist on artificial devices like mechanical ventilation equipment and catheters. Our study identified prolonged mechanical ventilation and urinary catheter use as independent risk factors for XDRAB infection. Mechanical ventilation compromises normal physiological function, connecting the airway to the external environment, destroying the weaker defense barrier, and allowing colonized XDRAB to enter the lower respiratory tract, leading to infection (Zilberberg et al., 2016; de Carvalho Baptista et al., 2018). Due to the drug resistance of XDRAB, the infection is difficult to control, leading to a vicious cycle, increased difficulty in weaning patients off mechanical ventilation, and even a higher risk of death (Huang et al., 2018). Indwelling urinary catheters, commonly used in clinical treatment for postoperative patients and those with incontinence or urinary retention, also facilitate AB colonization and the development of drug resistance. Meta-analyses, A recent meta-analysis identified the use of urinary catheters as one of the risk factors for XDRAB infection (Deshwal et al., 2023).

Among the underlying diseases in this cohort, including cerebrovascular disease, cardiovascular disease, chronic pneumonia, hypertension, and diabetes, only diabetes was a risk factor for XDRAB infection. Diabetes is often accompanied by complications and disturbances in humoral immunity, such as a reduced response of complement factor 4 and cytokines after stimulation. Additionally, studies have shown that the function of polymorphonuclear cells and monocytes/macrophages (chemotaxis, phagocytosis, and killing) is impaired in diabetics. High glucose environments also enhance the virulence and adherence of certain pathogens (Geerlings and Hoepelman, 1999). Diabetic patients often face impaired blood perfusion, reducing the effectiveness of antimicrobial drugs and facilitating the growth of drug-resistant bacteria. A report from Malaysia indicated that Chinese diabetic patients are more susceptible to CRAB infections and face a higher risk of death, potentially due to genetic factors that make them more prone to severe diseases compared to other ethnic groups (Woon et al., 2021). Many diabetic patients who initially respond to antimicrobial treatment for MDRAB are found to be reinfected with drug-resistant bacteria within a month (Lin et al., 2016). Ching-Hsiang Leung’s team reported that hypoglycemia is a risk factor for increased mortality in CRAB-infected patients and emphasized the importance of glycemic control, noting that insulin therapy is a major cause of hypoglycemia (Leung and Liu, 2019). Interestingly, a recent study found AB presence in the serum of 23% of tested type 2 diabetic patients (Perera et al., 2021). There remains a need for more prospective studies in the areas of glycemic control and XDRAB infection to further validate these findings.

For ICU patients or other high-risk individuals, prophylactic use of antimicrobial drugs is common, often involving combinations of these medications. However, the irrational use of antibiotics and their inherent toxicity can disrupt the patient’s microbiota, leading to the emergence of bacterial resistance, invasion by colonizing bacteria, and replacement of sensitive bacteria with resistant strains. This also results in decreased immunity and increased risk of infection by resistant pathogens (Bassetti et al., 2018). Our study identified the prophylactic use of carbapenems (such as imipenem and meropenem) and β-lactamase inhibitors (such as piperacillin/tazobactam and cefoperazone/sulbactam) as independent risk factors for XDRAB infection. Treating AB infections is particularly challenging in distinguishing between acute infection and upper respiratory tract colonization in critically ill patients, especially those with severe immunodeficiency or other high-risk factors. Consequently, empirical treatment for AB is often initiated when the clinical diagnosis is unclear (Bartal et al., 2022).

Sensitivity thresholds for some antimicrobials against XDRAB vary between CLSI and EUCAST. Currently, the isolation rates of CRAB worldwide range from 30% to 80%, with higher rates generally observed in Asian countries (Seifert et al., 2022). Studies have shown that once resistance to carbapenems develops, resistance to many other antimicrobials often follows (Zhang et al., 2017). XDRAB resistance to carbapenems is usually associated with the horizontal gene transfer of oxacillinase (OXA) genes, particularly OXA-23 and OXA-24/40.

Piperacillin-tazobactam, often used empirically due to its pharmacodynamic efficacy when administered in high doses with extended infusions, remains effective against *E. coli*, *K. pneumoniae*, and *P. aeruginosa*. However, it is ineffective against *A. baumannii* due to high resistance rates, and frequent empirical use can select for highly resistant strains. Sulbactam, primarily a β-lactamase inhibitor, is typically combined with β-lactam antibiotics to prevent enzymatic degradation. Nevertheless, increasing resistance to cefoperazone/sulbactam in *A. baumannii* has been observed. Therefore, the rational use of antimicrobials, especially carbapenems and β-lactamase inhibitors, remains crucial.

Antimicrobial use is generally guided by bacterial culture and sensitivity results, but traditional identification methods are time-consuming. Emerging high-throughput sequencing technologies, such as Next-Generation Sequencing (NGS), can rapidly detect and characterize pathogens. Although NGS offers significant advantages, its high cost currently limits widespread clinical adoption (Wen et al., 2020).

Our study utilized LASSO regression analysis to screen clinical variables, effectively reducing data dimensionality and preventing multicollinearity among independent variables. This approach addresses the limitations of retrospective studies, which are often constrained by sample size and cannot include a large number of variables. As a visualization method for predictive models, nomograms have become increasingly popular in clinical research. The factors influencing XDRAB infection are complex and interrelated. The predictive model developed in this study, based on eight independent risk factors identified through regression analysis, is user-friendly and allows for quantitative risk assessment. This aids clinicians in quickly assessing the risk of XDRAB infection using existing clinical data and implementing timely isolation and treatment measures. Moreover, this study employed stringent inclusion and exclusion criteria, ensuring a sufficient sample size and relevant factors. The nomogram underwent internal and external validation, demonstrating excellent discrimination, calibration, and strong clinical utility and stability.

However, this study has certain limitations. Firstly, being a single-center study, the model’s coefficients might vary under different circumstances, introducing geographic bias and limiting generalizability. External validation with datasets from other healthcare institutions, regions, and countries is necessary. Secondly, as a retrospective study, the data were obtained from electronic medical records and laboratory systems, which could result in some data being incomplete, and some factors that we intended to include in the statistical analysis could not be included. Selection bias is also unavoidable. Future prospective studies are needed to further validate these findings.

## 5 Conclusion

In this study, we identified that ICU length of stay, hypoproteinemia, glucocorticoid use, urinary catheters, mechanical ventilation, diabetes mellitus, carbapenem use, and β-lactamase inhibitor use were significantly correlated with XDRAB infection. Using these eight indicators, we developed a nomogram for individualized and visualized prediction of XDRAB infection risk in hospitalized patients. This predictive model facilitates early identification and mitigation of avoidable risks, allows for timely isolation and treatment of high-risk patients, and guides clinicians in rational antibiotic use. These findings provide valuable insights for improving the prevention and control of hospital-associated infections.

## Data Availability

The original contributions presented in the study are included in the article/supplementary material. Further inquiries can be directed to the corresponding author/s.
